# T-cell acute lymphoid leukemia resembling Burkitt leukemia cell morphology: A case report

**DOI:** 10.3892/ol.2014.2825

**Published:** 2014-12-23

**Authors:** QINGFANG YUE, XINYUE LIU, LEI CHEN, ZHONGPING LIU, WANXIN CHEN

**Affiliations:** Department of Hematology, Union Hospital, Tongji Medical College of Huazhong University of Science and Technology, Wuhan, Hubei 430022, P.R. China

**Keywords:** biphenotypic acute leukemia, acute lymphoblastic leukemia, Burkitt leukemia

## Abstract

Biphenotypic acute leukemia (BAL) is an uncommon type of cancer, which accounts for <5% of all adult ALs. Based upon a previously described scoring system, the European Group for the Immunological Classification of Leukemias (EGIL) proposed a set of diagnostic criteria for BAL. This scoring system is based upon the number and degree of specificity of several markers for myeloid or T/B-lymphoid blasts. The present study describes a case of T-cell acute lymphoblastic leukemia (T-ALL) with Burkitt-like cytology, which according to the French-American-British classification, corresponded to a diagnosis of Burkitt type L3 ALL. Flow cytometry analysis demonstrated that the blasts were positive for T-lymphoid markers, cytoplasmic cluster of differentiation (CD)3, CD7 and CD56, and myeloid markers, CD13, CD33 and CD15. At first, a diagnosis of BAL was suggested by the EGIL score, however, according to the 2008 World Health Organization criteria, a case of T-ALL with aberrant myeloid markers was established. The study also reviewed the literature and discussed the limitations of the EGIL scoring system in clinical decision making, to aid in the selection of an appropriate therapeutic regimen.

## Introduction

The majority of cases of acute leukemia (AL) originate from a specific cellular lineage, either lymphoid or myeloid, and can therefore be classified as acute lymphoblastic leukemia (ALL) or acute myeloid leukemia (AML). This classification is based upon morphological features and the cytochemical and immunophenotypical profile of the blast cells (1). In a small number of cases, the leukemic cells express markers belonging to more than one lineage. This may include two separate blast populations, one myeloid and one lymphoid, or a single blast population that expresses myeloid and lymphoid markers simultaneously (2). According to the most recent World Health Organization (WHO) classification (3), these types are considered as AL of ambiguous lineage. Biphenotypic AL (BAL) represents ~5% of adult AL cases, and is defined as a single blast population that co-expresses two lineage markers (4,5).

Burkitt leukemia (BL) is a highly-aggressive, mature B-cell neoplasm, which is classified as an L3 ALL by the French-American-British system (6). BL exhibits unique morphological characteristics and has the cytogenetic hallmark of t(8;14)(q24;q32) (7). The present study describes a rare case of T-cell ALL (T-ALL), with cells co-expressing myeloid markers, which highly resembled the classic Burkitt leukemia morphology.

## Case report

**Figure 1:**
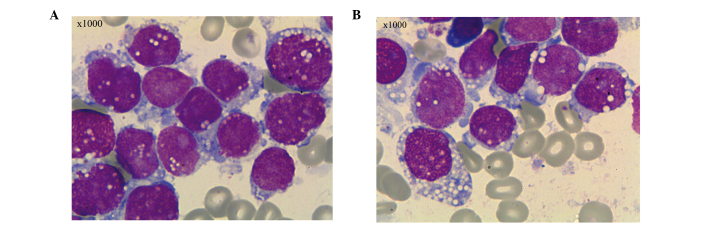
(A and B) Two representative images of the patient bone marrow smears with Wright-Giemsa stain revealing the presence of Burkitt-like cells (magnification, ×1,000).

On June 9, 2013, a previously healthy 37-year-old male was admitted to a local hospital with palpitations and night sweats that had been apparent for one month. A complete blood count (CBC) revealed a white blood cell (WBC) count of 2.89×10^9^/l (normal range, 4–10×10^9^/l), a red blood cell count (RBC) of 1.43×10^12^/l (normal range, 4.4–5.5×10^12^/l), a hemoglobin (Hb) level of 46 g/l (normal range, 120–160 g/l) and a platelet (PLT) count of 91×10^9^/l (normal range, 100–300×10^9^/l). A bone marrow aspiration identified significantly increased numbers of raw and immature lymphocytes, medium-sized cells containing a large number of lipid vacuoles with abundant, basophilic cytoplasm, and round nuclei with clumped chromatin and multiple nucleoli. According to the morphological bone marrow features, a diagnosis of Burkitt leukemia was established. Therefore, an RBC transfusion and vitamin 12 supplements were administered. After five days, the patient was referred to the Department of Hematology, Union Hospital, Tongji Medical University of Science and Technology (Wuhan, China) for further observation. A CBC revealed a WBC count of 5.24×10^9^/l, a RBC count of 1.82×10^12^/l, an Hb level of 59 g/l and a PLT count of 136×10^9^/l. The bone marrow and peripheral blood smear revealed 96% and 25% blast cells, respectively. The blast cells were variable in size, contained moderate to abundant amounts of cytoplasm and were stained blue. Certain cells exhibited pseudopodia-like protrusions, but the majority contained immature lacy chromatin, marked nucleoli, and cytoplasm consisting of abundant lipid vacuoles and granules. The features of the cells highly resembled those of Burkitt leukemia cells (Fig. 1). Cytochemical staining revealed that the cells were negative for myeloid peroxidase (MPO), acid α-naphthyl acetate esterase (ANAE) and chloroacetate esterase, but positive for periodic acid-Schiff.

Immunophenotyping detected a single blast population with distinct immunoreactivity for T-lymphoid and myeloid antigens. The blasts expressed cluster of differentiation (CD) 3, 7, 13, 11b, 33, 34, 56 and 38, and human leukocyte antigen-DR. There was no significant evidence of mature T and B cells in the background population. G-banding analysis revealed metaphase cells with normal karyotypes.

The patient received induction chemotherapy with hyper-Cytoxan (300 mg/m^2^, i.v., every 12 h, days 1–3), vincristine (2 mg, i.v., days 4 and 11), Adriamycin (50 mg/m^2^, i.v., day 4), dexamethasone (40 mg/day, i.v., days 1–4 and days 11–14) and Ara-C (70 mg, intrathecal, day 7). Following once cycle of chemotherapy, the bone marrow aspirate was reevaluated, which revealed no evidence of leukemia. The patient attained complete morphological remission, but subsequently succumbed due to severe complications following a stem cell transplantation procedure. Written informed consent was obtained from the patient’s family for the publication of this study.

## Discussion

The present study reports a rare case of adult AL, in which the blast cells co-expressed T-lymphoid and myeloid antigens, and in which the classical morphological features of Burkitt leukemia were apparent. BAL is a rare disease, which refers to a form of AL with a single population of blasts that co-express markers of two different lineages (4), BAL is often confused with acute bilineal leukemia, which is composed of a mixed population of leukemic cells originating from two distinct lineages (8–10). In 1995, a consensus criteria for the diagnosis of BAL was established by the European Group for the Immunological Characterization of Leukemias (EGIL) to distinguish between patients with BAL and those with AL with an aberrant expression of markers from a different lineage. Within this scoring system, CD markers are assigned a score of 0.5, 1.0 or 2.0, depending upon whether a particular antigen originates from a myeloid, B- or T-lymphoid lineage (Table I) (11). According to the EGIL, a score of >2 points is sufficient to assign membership in a cell line. If the score is <2 for the other cell lines, or other markers of these lines, they should be qualified as aberrant markers. Using this system, a diagnosis of T-ALL/AML can be established.

The 2008 WHO classification of hematological tumors adopted the EGIL criteria for BAL and introduced a novel group of AL termed ‘AL of ambiguous lineage’. However, the 2008 WHO classification adopts a more restrictive criteria than the EGIL to define BAL (12). According to the 2008 WHO criteria, BAL originates from a lineage of cells that express MPO, CD19 and cytoplasmic CD3 (3). In the present study, MPO expression was negative, and therefore, the likely diagnosis was T-ALL with aberrant expression of myeloid markers. Suggs *et al* (13) reported that the myeloid markers CD13 and CD33 are commonly expressed in certain precursor T-ALLs.

Coche *et al* (14) described a case of BAL with Burkitt-like morphology, in which the cells co-expressed the myeloid markers, IgM, CD79a, 19, 22 and 24, and the B-lymphoid lineage markers, CD13, 33, 65 and 15. To the best of our knowledge, the present study was the first to describe a case of T-lymphoid and myeloid lineage marker co-expression with Burkitt-like cells. The patient was initially misdiagnosed at the local hospital due to the atypical cellular morphology, however, the clinical features of the patient differed from those of the majority of BL cases. Firstly, BL is a mature B-cell neoplasm. The immunological phenotype in the present case was markedly different from BL. Secondly, 80% of BL cases harbor the t(8;14) translocation; in the remaining 20% of cases, translocations exist between chromosomes 2 and 8, t(2;8)(p12;q24), or between chromosomes 8 and 22, t(8;22)(q24;q11) (15–17). By contrast, the cytogenetic profile of the patient in the present study appeared normal.

Of the scoring systems used for the classification of BAL, EGIL is usually applied during routine clinical practice. However, certain limitations of this classification system exist. Firstly, the EGIL does not define lineage-specific markers. Certain lineage-specific markers, such as CD3, CD22 and MPO, may be only slightly higher than lineage-associated markers, such as CD7, 13, 19, 20 and 33. This has the potential to lead to an overdiagnosis of BAL. Secondly, the EGIL is based upon immunological markers and omitted cytogenetic data, therefore, even well-defined AML may be misdiagnosed as BAL. Finally, as there are no standard treatment regimens for BAL, the EGIL is unable to predict optimal therapy decisions. Therefore, hematologists/oncologists may choose to treat patients with regimens for either AML or ALL, or both. Improved lineage definition will provide clinicians with suitable guidelines for selecting appropriate therapeutic regimens, since the treatment of AML differs from that of ALL.

At present, there is no uniform agreement regarding the treatment of patients with BAL. According to the literature (9,18), ALL-oriented chemotherapy exhibits a higher complete remission rate compared with AML-oriented chemotherapy. However, according to the 2008 WHO classification, the present case was more inclined to be T-ALL with aberrant myeloid makers, and therefore, the patient was administrated ALL-based induction chemotherapy. Despite progress in the treatment of AL (8,19), the prognosis of BAL or T-ALL with myeloid markers, and the response to drug therapies that target conventional ALL, remains poor.

To the best of our knowledge, the present study was the first to report a case of T-ALL with cells co-expressing myeloid makers and highly resembling the classic morphology of Burkitt leukemia cells. The diagnosis was determined by the 2008 WHO classification, and not the EGIL. This case demonstrated the heterogeneity of AL, not only in regard to cellular morphology, but also immunological performance.

## Figures and Tables

**Table I:** European Group for the Immunological Classification of Leukemias scoring system.

Points	B-lymphoid lineage	T-lymphoid lineage	Myeloid lineage
2	CytCD79a	CD3	MPO
	CytIgM	anti-TCR	
	CytCD22		
1	CD19	CD2	CD117
	CD20	CD5	CD13
	CD10	CD8	CD33
		CD10	CD65
0.5	TdT	TdT	CD14
	CD24	CD7	CD15
		CD1a	CD64

Cyt, cytoplasmic; CD, cluster of differentiation; IgM, immunoglobulin M; TCR, T-cell receptor; MPO, myeloid peroxidase; TdT, terminal deoxynucleotidyl transferase.
